# An Immunosensor Based on Antibody Binding Fragments Attached to Gold Nanoparticles for the Detection of Peptides Derived from Avian Influenza Hemagglutinin H5

**DOI:** 10.3390/s140915714

**Published:** 2014-08-25

**Authors:** Urszula Jarocka, Róża Sawicka, Anna Góra-Sochacka, Agnieszka Sirko, Włodzimierz Zagórski-Ostoja, Jerzy Radecki, Hanna Radecka

**Affiliations:** 1 Institute of Animal Reproduction and Food Research of Polish Academy of Sciences, Tuwima 10, 10-748 Olsztyn, Poland; E-Mails: u.jarocka@pan.olsztyn.pl (U.J.); j.radecki@pan.olsztyn.pl (J.R.); 2 Institute of Biochemistry and Biophysics, Polish Academy of Sciences, Pawińskiego 5A, 02-106 Warsaw, Poland; E-Mails: rozasawicka@ibb.waw.pl (R.S.); annag@ibb.waw.pl (A.G.-S.); asirko@ibb.waw.pl (A.S.); wlodzimierz.zagorski@ibb.waw.pl (W.Z.-O.)

**Keywords:** avian influenza virus, Fab' fragments, gold electrodes, electrochemical immunosensor, electrochemical impedance spectroscopy

## Abstract

This paper concerns the development of an immunosensor for detection of peptides derived from avian influenza hemagglutinin H5. Its preparation consists of successive gold electrode modification steps: (i) modification with 1,6-hexanedithiol and gold colloidal nanoparticles; (ii) immobilization of antibody-binding fragments (Fab') of anti-hemagglutinin H5 monoclonal antibodies Mab 6-9-1 via S-Au covalent bonds; and (iii) covering the remaining free space on the electrode surfaces with bovine serum albumin. The interactions between Fab' fragments and hemagglutinin (HA) variants have been explored with electrochemical impedance spectroscopy (EIS) in the presence of [Fe(CN)_6_]^3−/4−^ as an electroactive marker. The immunosensor was able to recognize three different His-tagged variants of recombinant hemagglutinin from H5N1 viruses: H1 subunit (17–340 residues) of A/swan/Poland/305-135V08/2006, the long HA (17–530 residues) A/Bar-headed Goose/Qinghai/12/2005 and H1 subunit (1–345 residues) of A/Vietnam/1194/2004. The strongest response has been observed for the long variant with detection limit of 2.2 pg/mL and dynamic range from 4.0 to 20.0 pg/mL.

## 1. Introduction

Avian influenza (AI) can be an extremely contagious disease of birds caused by type A influenza virus, a member of the family *Orthomyxoviridae*. It is an enveloped RNA virus about 100 nm in diameter with an eight-segmented, single-stranded and negative-sense genome. Two glycoproteins, hemagglutinin (HA) and neuraminidase (NA), are present on the surface and play important roles in infecting a host cell. Sixteen HA (H1–H16) and nine NA (N1–N9) subtypes of the influenza virus are found [1,2].

Influenza A is the only virus type that can infect birds, and almost all wild life birds and domestic poultry can be infected. Highly pathogenic variants of avian influenza (HPAI) virus often cause systemic infections in poultry that result in huge economic losses around the world [3]. The H5N1 HPAIV virus has been shown to spread incessantly in many regions all over the world [4]. Most of these outbreaks were confined to poultry, but the virus was reported to be transmitted to humans in a few countries and this often lead to deaths in infected humans [5]. Consequently, rapid and sensitive technology for detection of H5N1 is urgently needed to help containing the spread of H5N1.

The traditional methods for avian influenza virus diagnostic are Enzyme Linked Immunosorbent Assay (ELISA) [2,6]. Reverse Transcriptase Polymerase Chain Reaction (RT–PCR) [7–9] and real-time electrical detection [10]. These methods are expensive and require highly trained laboratory staff. Immunosensors provide a promising alternative to currently used detection systems [11–13]. They are analytical devices consisting of antibodies or their fragments coupled to a transducer and they generate an analytical response related to analyte concentration in a sample [14,15].

Antibodies are considered to be the first choice as molecular recognition elements due to their target specificity and affinity, which make them excellent probes in biosensor development. These biological molecules are special kinds of proteins made by cells of the immune system, B-lymphocytes. The antibody molecule shaped as “Y” consists of two identical polypeptide chains (Fab' fragments) responsible for antigen binding, and one Fc domain. The Fc fragment could be removed by enzyme digestions [16–18]. The prepared F(ab')_2_ or Fab' fragments could be self-assembled on the gold surface or other functionalized group due to disulfide or thiol group from the hinge region of the antibody (immunoglobulin G). The covalent immobilization is stronger in comparison to electrostatic interactions between antibodies and sensing platform. In addition, covalent bonds enable appropriate orientation and accessibility of antibody binding sites [19–21].

Immunosensors have found widespread applications in food industry, environmental and pollutants, biotechnology, pharmaceutical chemistry and clinical diagnostics [22–27]. Several types of immunosensors, such as surface plasmon resonance (SPR) [3,28,29] and quartz crystal microbalance (QCM) [30–34], optical interferometric [35,36], label-free microcantilever [37] and fluorescent [38] have been researched as alternatives to the conventional influenza virus detection methods. These biosensors have shown analytical potential, but they lack subtype specificity. Many of them are also not practical for use in the field. Compared to these devices electrochemical biosensors [39,40] appear very promising because they require minimal instrumentation and are readily integrated with microelectronics. Among electrochemical immunosensors, electrochemical impedance spectroscopy (EIS)-based label-free sensing has gained much attention [11,13,19,25,41–45]. Impedance immunosensors have advantages over traditional influenza virus detection methods due to their simple design and relatively low cost. EIS is a very powerful non-destructive method and can be employed to study a biological interaction. In this method data points are generated using a small perturbation in that reduces the matrix interference coming from the composition of biological samples. Several successful applications of EIS for detection of different biomolecules such as antibodies [46], His-tagged proteins [19,25], viruses [47–49] and Aβ peptide [50,51] have been reported by our group.

Here, we present a sensitive immunosensor for the detection of peptides derived from avian influenza hemagglutinin H5 based on the recognition of the epitope localized in the N-terminus of HA by polypeptide Fab' from monoclonic Mab 6-9-1. Specific exposure of thiol groups by the Fab' fragment allows for random dense and precise saturation of the sensor surface with antibodies. This simplifies the preparation of electrode surfaces and sensing of the HA antigen composition. Specific interactions between oriented Fab' fragments immobilized on the gold electrode surface and three different His-tagged variants of recombinant hemagglutinin: H1 subunit (17–340 residues) of A/swan/Poland/305-135V08/2006 (Clade 2.2), H1 subunit (1–345 residues) of A/Vietnam/1194/2004 (Clade 1) and the truncated HA (without signal peptide and the transmembrane domain; 17–530 residues) A/Bar-headed Goose/Qinghai/12/2005 (Clade 2.2) were observed using EIS in the presence of [Fe(CN)_6_]^3−/4−^ as an electroactive marker.

## 2. Experimental Section

### 2.1. Chemicals and Preparations

Gold colloidal nanoparticles (17–23 nm in diameter), 1,6-hexanedithiol (1,6-HDT), potassium ferro- and ferricyanides, phosphate buffer saline (PBS) components (137 mM NaCl, 2.7 mM KCl, 1.8 mM Na_2_HPO_4_, 10 mM KH_2_PO_4_) were supplied by Sigma-Aldrich (Poznań, Poland). Alumina slurry 0.3 and 0.05 μm was purchased from Buehler (Lake Bluff, IL, USA). Sulphuric acid, potassium hydroxide, ethanol and methanol were obtained from POCh (Gliwice, Poland). Bovine serum albumin (BSA) was purchased from Invitrogen Life Technologies (Darmstadt, Germany). Hybridoma producing anti-hemagglutinin H5 monoclonal antibodies (Mab 6-9-1) was from the Institute of Biotechnology and Antibiotics (Warsaw, Poland). The Mab 6-9-1 antibodies were purified using the NAb Protein G Spin Kit according to manufacturer protocol (Pierce, Rockford, IL, USA) and the Fab' fragments were digested by papain agarose from *Papaya latex* (Sigma Aldrich, P4406-25UN) with the 1:2 (weight to weight) ratio of papain agarose to Mab 6-9-1. Efficiency of digestion was verified by western blot analysis where Fab' fragments were detected by goat anti mouse IgG Fab' specific antibody. Three His-tagged recombinant hemagglutinin variants of H5N1 virus were used in this work: (i) HA/Nde, (ii) Qinghai and (iii) Vietnam. The “HA/Nde” protein is based on the sequence of A/swan/Poland/305-135V08/2006 (EpiFlu Database Acc no. EPI156789) and covers region of 17–340 residues (corresponding to the H1 subunit). The “HA/Nde” protein was produced in*Escherichia coli* cells in fusion with 6xHis-tag and affinity purified. The “Qinghai” protein (based on the sequence of A/bar headed goose/Qinghai/12/2005; 17–530 residues) and the “Vietnam” protein (based on the sequence of A/Vietnam/1194/2004; 1–345 residues) were produced in mammalian cells and purchased from Immune Technology (New York, NY, USA).

All aqueous solutions were prepared using Milli-Q water, resistivity 18.2 MΩ·cm (Millipore, Darmstadt, Germany). Reagents and solvents were of analytical grade and were used without further purification. Experiments were carried out at room temperature unless stated otherwise.

### 2.2. Preparation of Immunosensor

The gold disk electrodes (2 mm diameter) were obtained from Bioanalytical System (BAS, West Lafayette, IN, USA). Electrodes after washing with methanol and Milli-Q water were polished in alumina slurries (Alpha and Gamma Micropolish, Buehler) with particles size of 0.3 and 0.05 μm on microcloth polishing pads (BAS) for 5 min each. Afterwards they were carefully washed with Milli-Q water. Then, electrochemical cleaning was performed by cyclic voltammetry (CV). At first they were dipped in 0.5 M potassium hydroxide solution and swept with a potential between −0.4 V and −1.2 V against the silver chloride reference electrode (Ag/AgCl) and the platinum wire counter electrode with a scan rate of 100 mV/s, number of cycles: 3, 50 and 10. Next, the electrodes were cleaned in 0.5 M sulphuric acid solution in the potential window between −0.3 V and +1.5 V, number of cycles: 3, 10 and 3. Before modification, the surfaces of electrodes were refreshed in 0.5 M potassium hydroxide solution for 10 cycles. After finishing the electrochemical cleaning, each electrode was rinsed with Mili-Q water and stored in water (for several minutes, until the next step) to avoid contaminations from air. All solutions were deoxygenated by purging with nitrogen (ultra pure 6.0, Air Products, Warszawa, Poland) for 10 min.

The clean gold electrodes were washed repeatedly with water and ethanol. Then, they were immersed for 20 h in 10 mM 1,6-hexanedithiol (1,6-HDT) solution in ethanol. The tubes containing electrodes and 1,6-HDT solution were sealed with Teflon tape and Parafilm to avoid solvent evaporation. Subsequently electrodes were rinsed with ethanol and water. Electrodes with formed 1,6-HDT self-assembled monolayer (SAM) were fixed upside down and a 10 μL droplets of gold colloidal nanoparticles (GCP) solution were spotted on each gold surface. The tubes containing electrodes were sealed with Parafilm and stored in +4 °C for 18 h. After incubation, electrodes were rinsed with water and 0.1 M phosphate buffer saline pH 7.4. Next 10 μL droplets of 1 μg/mL Fab' 6-9-1 in PBS buffer were aliquoted onto the surface of each electrode. The tubes with electrodes were again sealed with parafilm and incubated in +4 °C for 20 h. Then, electrodes were carefully rinsed with PBS buffer. Bovine serum albumin (BSA) solution (in 0.1 M PBS pH 7.4) in concentration of 0.5% (mass/volume) was used for blocking of unspecific binding. As in prior steps, a 10 μL droplets were spotted on each electrode and stored in +4 °C for 2 h. Finally, electrodes were rinsed with 0.1 M PBS. Fully modified electrodes were kept in refrigerator (+4 °C) in 0.1 M PBS buffer pH 7.4 until use, no longer than one day.

### 2.3. Electrochemical Measurements

All electrochemical measurements were performed at room temperature with an AutoLab potentiostat-galvanostat (Eco Chemie, Utrecht, The Netherlands) using a three-electrode configuration. Working electrodes were polycrystalline gold discs 2 mm diameter (BioAnalytical System). All potentials were measured versus an Ag/AgCl reference electrode and platinum wire was used as the counter electrode. Cyclic voltammetry (CV) and electrochemical impedance spectroscopy (EIS) were performed in solution comprised of 0.1 M PBS pH 7.4 and K_3_[Fe(CN)_6_]/K_4_[Fe(CN)_6_] (0.5 mM each). In the CV potential were cycled from 0.6 to −0.2 V with scan rate 0.1 V/s. The EIS were recorded within the frequency range of 0.1 Hz to 10 kHz at formal potential of the redox couple [Fe(CN)_6_]^3−/4−^ (0.17 V) with ac amplitude of 10 mV. Obtained spectra were fitted by the AutoLab software in order to obtain values of electron transfer resistance (*R_i_*). The electrode responses were expressed as: (*R_i_*−*R*_0_)/*R*_0_ where R_0_ means electron transfer resistance of fully modified electrode measured in pure PBS buffer before His-tagged recombinant proteins detection, *R_i_* means electron transfer resistance of fully modified electrode measured in PBS containing particular concentration of the detected proteins.

## 3. Results and Discussion

### 3.1. Immunosensor Preparation and Its Electrochemical Characterization

**Figure 1.**
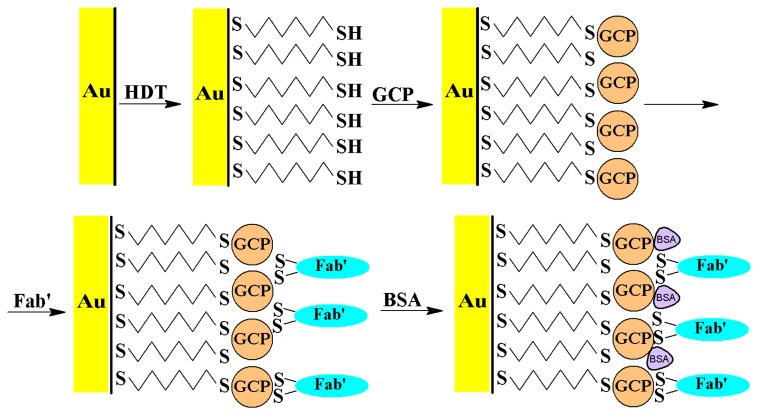
Schematic illustration of the process of immobilization of Fab' fragments onto the gold electrode surface.

The process of immunosensor fabrication is shown in Figure 1. After cleaning gold electrodes were coated with 1,6-hexanedithiol. Next, a colloidal gold nanoparticles layer is formed via covalent Au-S bonds. Fab' 6-9-1 was deposited onto the colloidal gold layer through covalent bonds between the Au atoms of the gold colloidal nanoparticles and the SH group of Fab' fragments. Bovine serum albumin was used to block the remaining spaces on the gold layer.

**Figure 2.**
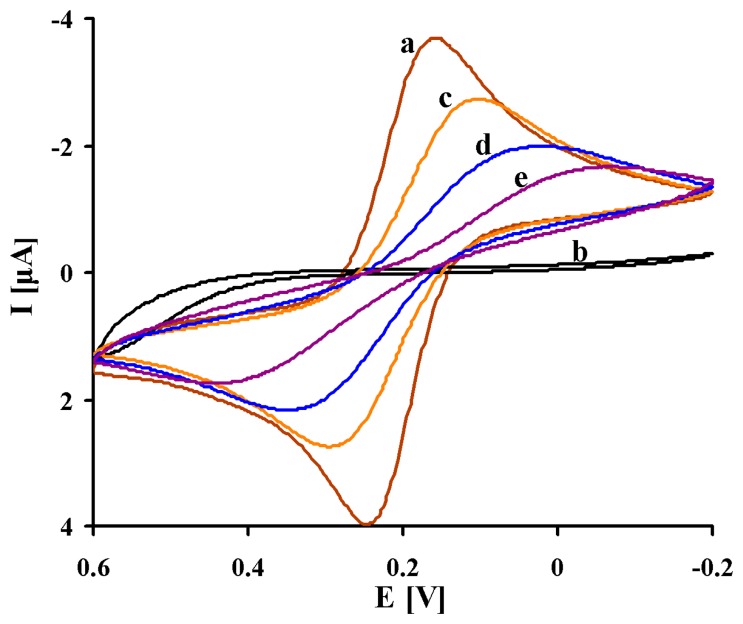
The typical cyclic voltammograms of: (a) bare gold electrode; (b) 1,6-HDT modified electrode; (c) GCP/1,6-HDT modified electrode; (d) Fab'/GCP/1,6-HDT modified electrode; (e) BSA/Fab'/GCP/1,6-HDT modified electrode. Solution composition: 1 mM K_3_[Fe(CN)_6_]/K_4_[Fe(CN)_6_], 0.1 M PBS pH 7.4. The measuring conditions: three electrode configurations—Au working electrode, Ag/AgCl reference electrode, and Pt counter electrode; scan rate 100 mV/s.

The cyclic voltammograms of each steps of modification were shown in Figure 2. Bare gold electrode in 1 mM K_3_[Fe(CN)_6_]/K_4_[Fe(CN)_6_] as redox probe in PBS showed a couple of redox peaks with Δ*E*_p_ = 75 mV (Figure 2, Curve a). After the covalent attachment of 1,6-HDT, shape of CV changed dramatically. The 1,6-HDT formed a self-assembled monolayer on the gold electrode surface. This results in blocking of the surface for the redox marker as can be seen in the CV with no apparent peaks (Figure 2, Curve b). The reversibility of the system was restored after immobilization of colloidal gold on the layer of 1,6-HDT with peak separation Δ*E*_p_ = 166 mV (Figure 2, Curve c). The colloidal gold particles (GCP) size (17–23 nm) have been selected and characterized by Atomic Force Microscopy in our previous study [49]. GCP increased the conduction pathways and promote the electron transfer between the redox marker and electrode surface [19,25,47,49–51]. The immobilization of Fab' fragments on the colloidal gold layer, formed the insulating layer and decreased the accessibility of [Fe(CN)_6_]^3−/4−^. This caused an increase of the CV peak separation to Δ*E*_p_ = 328 mV (Figure 2, Curve d). Further decreasing of faradaic current was observed upon immobilization of BSA onto the remaining sites on the gold layer (Figure 2, Curve e).

**Figure 3.**
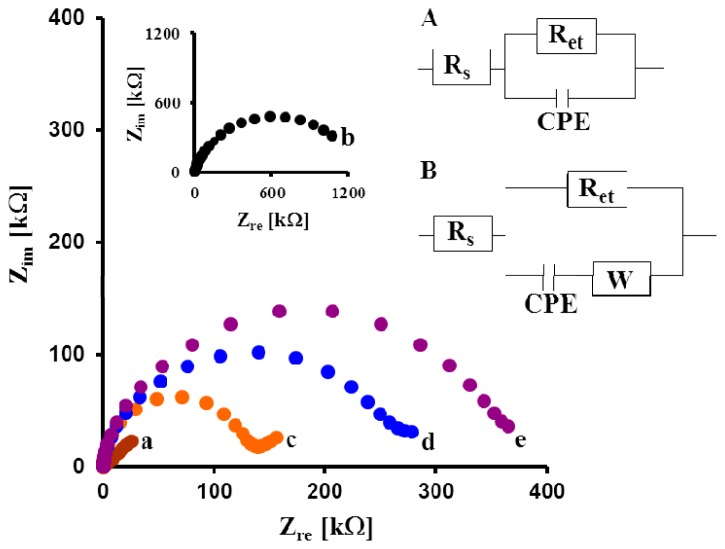
The typical electrochemical impedance spectra of: (a) bare gold electrode; (b) 1,6-HDT modified electrode; (c) GCP/1,6-HDT; (d) Fab'/GCP/1,6-HDT modified electrode; (e) BSA/Fab'/GCP/1,6-HDT modified electrode. Solution composition: 1 mM K_3_[Fe(CN)_6_]/K_4_[Fe(CN)_6_], 0.1 M PBS pH 7.4. The measuring conditions: three electrode configurations—Au working electrode, Ag/AgCl reference electrode, and Pt counter electrode; a bias potential of 0.17 V; the frequency range from 0.1 Hz to 10 kHz. Inset: circuit models used for fitting Nyquist plots for: (**A**) Curves b and e, (**B**) Curves c and d. *R_s_*—solution resistance, *R_et_*—electron transfer resistance and CPE—constant phase element, W—Warburg.

The each step of the gold electrode modification was also controlled using EIS. Impedance spectra are shown as Nyquist plots of real (*Z_re_*) *vs.* imaginary (*Z_im_*) impedance (Figure 3). The bare gold electrode exhibited an almost straight line in the Nyquist plot, which is characteristic of a diffusion-limited electrochemical process (Figure 3, Curve a). The 1,6-HDT modified electrode showed a semicircular plot at higher frequencies (Figure 3, Curve b). This indicated that the electrode redox processes were limited by electron transfer (*R_et_* = 1359.3 kΩ). The chemisorptions of GCP on the 1,6-HDT layer decreased the electron transfer resistance to 112.4 kΩ (Figure 3, Curve c). The immobilized Fab' fragments were covalently linked to the colloidal gold layer through Au-S bonds. This caused an increase of the electron transfer resistance to 303.1 kΩ (Figure 3, Curve d). Blocking remaining sites of colloidal gold layer with BSA increased the resistance to 459.4 kΩ (Figure 3, Curve e). The results obtained from EIS measurement (Figure 2) connected with each step of the electrode modification are in good agreement with the results of CV (Figure 3).

### 3.2. Immunosensor Electrochemical Impedance Responses towards His-Tagged Variants of H5 HA

**Figure 4.**
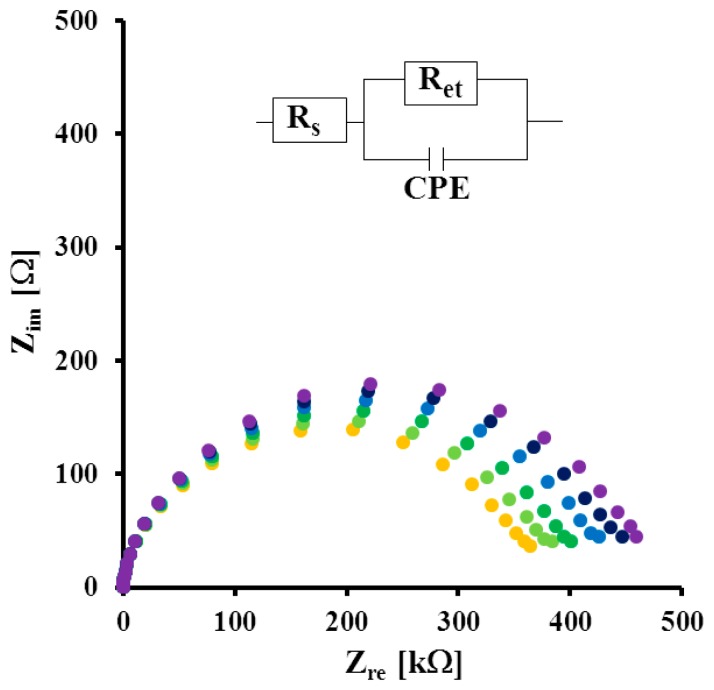
The typical electrochemical impedance spectra of BSA/Fab'/GCP/1,6-HDT modified electrode (a) in buffer solution; and after applying (b) 4 pg/mL; (c) 8 pg/mL; (d) 12 pg/mL; (e) 16 pg/mL; (f) 20 pg/mL of Qinghai polypeptide. Solution composition: 1 mM K_3_[Fe(CN)_6_]/K_4_[Fe(CN)_6_], 0.1 M PBS (pH 7.4). The measuring conditions: three electrode configurations—Au working electrode, Ag/AgCl reference electrode, and Pt counter electrode; a bias potential of 0.17 V; the frequency range: from 0.1 Hz to 10 kHz. Circuit model used for fitting Nyquist plots in inset: *R_s_*—solution resistance, *R_et_*—electron transfer resistance and CPE—constant phase element.

Quantitative assessment of the immunosensor sensitivity was performed using serial dilutions of three different His-tagged variants of recombinant H5 HA: HA/Nde, Qinghai and Vietnam in PBS buffer. The representative EISs recorded in the presence of Qinghai are illustrated in Figure 4. Electron transfer resistance measured for the immunosensor in pure PBS buffer *R*_0_ (Figure 4, Curve a) was used to calculate the relative response towards a specific fragment of virus. Interactions between Fab' fragments and Qinghai caused decreased accessibility of the redox marker [Fe(CN)_6_]^3−/4−^ to the gold electrode surface. As a result we observed a rise of the electron transfer resistance *R_i_* with addition of increasing concentrations of the antigen (Figure 4, Curves b–f).

**Figure 5.**
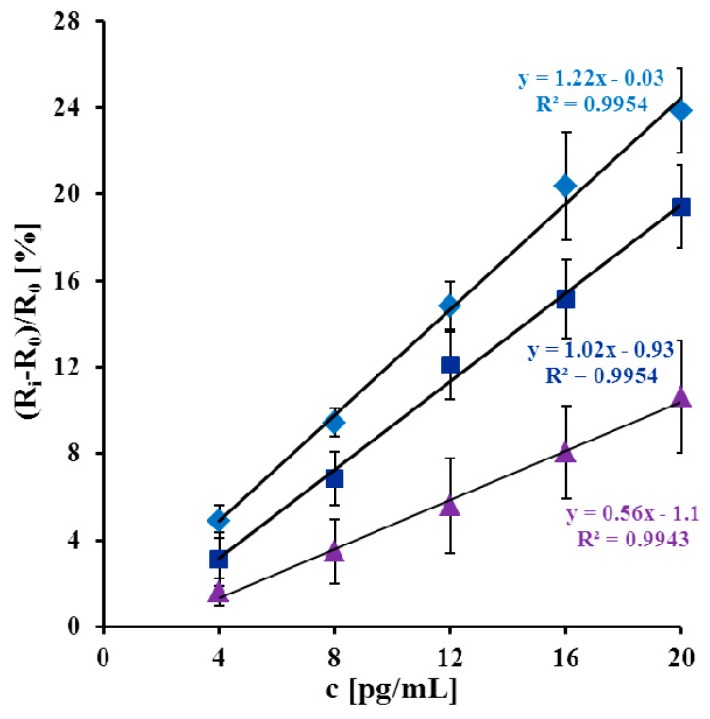
The relationship of (*R_i_* – *R*_0_)/*R*_0_ *vs.* concentrations [pg/mL] of: (◆) Qinghai; (■) HA/Nde and (▲) Vietnam polypeptide. *R*_0_ is the electron transfer resistance of fully modified electrode measured in pure PBS buffer before antigen application and *R_i_* is the electron transfer resistance of fully modified electrode measured in 0.1 M PBS buffer with the given concentration of antigen (n = 3/5).

The linear range of analytical response was from 4 to 20 pg/mL. This device was able to detect three different His-tagged proteins (Qinghai, HA/Nde and Vietnam) with different sensitivity. The highest concentration of antigen (20 pg/mL) caused a significant increase of the electron transfer resistance to 23.9 ± 2.0% for Qinghai, 19.4 ± 1.9% for HA/Nde and 10.3 ± 2.5% for Vietnam (Figure 5).

Limits of detection (LOD) were calculated based on the standard deviation of the response and the slope of the calibration curve:

(1) LOD=3.3σ/S

where σ is the standard deviation of the response and *S* is the slope of the calibration curve [52]. Limits of detection were 2.2 pg/mL for Qinghai, 4.0 pg/mL for HA/Nde and 3.5 pg/mL for Vietnam. Sensitivities of the proposed biosensor were 1.2%[(*R_i_* − *R*_0_)/*R*_0_c] pg/mL for Qinghai, 1.0%[(*R_i_* − *R*_0_)/*R*_0_c] pg/mL for HA/Nde and 0.6%[(*R_i_* − *R*_0_)/*R*_0_c] pg/mL for Vietnam. The immunosensor presented in this paper thus has good sensitivity. This is mainly because of the immobilization strategy used. The covalent bonds between Au atoms of GCP and SH group of Fab' fragments enable stable and appropriate orientation for antigen binding.

Parental monoclone Mab 6-9-1 is presumably directed toward a conformational epitope located within the H1 domain of the HA protein of H5N1 (A/swan/Poland/305-135V08/2006) virus. As expected, the sensor equipped with Fab' fragments of this monoclone efficiently recognizes the HA/Nde antigen representing H1 domain coded by homologous virus as well as Qinghai polypeptide coded by the virus from the same IV Clade 2.2.

The immunosensor shows lower affinity to Vietnam antigen expressing the similar H1 region, however coded for by the strain belonging to the distant H5N1 virus Clade 1. When calculating sensitivity in molar antigen concentrations, the proposed immunosensor shows the highest affinity toward Qinghai polypeptide equipped besides the H1 domain with further fragments of HA. This additional region (between aa 341-530) does not carry epitopes recognized by the parental Mab 6-9-1. The observed rise in sensitivity suggests that the additional HA polypeptide fragment is implicated in exposition of the H1 domain, confirming the claim that Mab 6-9-1 recognizes conformational epitope. The proposed sensor equipped with Fab' fragments follows the characteristics of the parental monoclone in distinguishing the conformers of the same epitope.

We are aware that final validation of the sensor requires testing its specificity against a collection of antigens from alien respiratory viruses. The characterisation of parental Mab 6-9-1 specificity is in progress (V. Sączyńska, personal communication) and this will lead to the requisite validation experiments.

In our the previous paper we created an immunosensor based on gold electrodes modified with 1,6-HDT, GCP, whole IgG antibodies and BSA for detection of His_6_-rSPI2 protein [19] and the Plum Pox Virus (PPV) with a 10 pg/mL limit of detection [49] (Table 1). The deposition of the whole antibodies on the colloidal gold layer relies on electrostatic interactions. Physical adsorption is one of the simplest protein binding processes, although rather uncontrollable. Random orientation of the absorbed antibodies and close proximity between adsorptive surface and the antigen-binding site could impede the detection. The immobilization of Fab for detection of His_6_-rSPI2 protein improves the detection limit to 5 pg/mL [25] in comparison to an immunosensor incorporating the whole IgG antibody [19], proving that this is right strategy for immunosensor design. The detection limit of the presented immunosensor in the range of 2.2 pg/mL is better in comparison to those already published [11,15,19,24,25,27,39,45] or at least at the similar level [40,41,46] (Table 1).

It is worth emphasizeing that the main advantages of the immunosensor proposed here are very small sample demand, good sensitivity and simple fabrication, with the possibility for miniaturization. The detection limits for nanoscale biosensors are mainly governed by analyte transport limitation towards sensing layers, not a signal transduction [53]. The sensor shape, analyte diffusing ability, as well as the appropriate analyte accumulation time are important parameters for nano-sensor design and will be taken into account in our future research.

## 4. Conclusions and Outlook

Concluding, a sensitive and selective impedimetric immunosensor for the detection of peptides derived from avian influenza hemagglutinin H5 using Fab' immobilized on a gold electrode surface via colloidal gold nanoparticles was developed. This device is able to recognize three different His-tagged fragments of HA: Qinghai, HA/Nde and Vietnam. The strongest response was observed for Qinghai, with a detection limit of 2.2 pg/mL and a dynamic range from 4.0 pg/mL to 20.0 pg/mL. The presented research shows that gold colloidal nanoparticles may be used for the creation of a very good underlayer for Fab' oriented immobilization. They allow fragments of antibodies to retain their activity and constitute a good electron conductive layer for electrochemical sensors. Considering its good selectivity and sensitivity in the pg/mL range, the proposed immunosensor was superior in comparison to others already reported, therefore, it could be recommended for the rapid, simple and direct electrochemical detection of avian influenza virus H5N1.

## Figures and Tables

**Table 1.** A comparison of detection limits of electrochemical sensors destined for antigen and antibody determination.

**No.**	**Electrode Modification**	**Measuring Technique**	**Analyte**	**Detection Limit**	**References**
1.	Au/ OT + BDDT/NV/Ab	EIS [Fe(CN)_6_]^3−/4−^	IAV	8 ng/mL	[11]
2.	Au/Protein A/Ab/BSA		H5N1 virus	10^3^ EID_50_/mL	[13]
3.	GCE/SiSG/Ab	CV, EIS [Fe(CN)_6_]^3−/4−^	Carbofuran	0.33 ng/mL	[15]
4.	Au/HDT/GNR/ Fab'/BSA	EIS [Fe(CN)_6_]^3−/4−^	rSPI2-His_6_	10 pg/mL	[19]
5.	Au/GNPs/Protein A/Ab/BSA	CV [Fe(CN)_6_]^3−/4−^	Carbofuran	0.1924 ng/mL	[24]
6.	Au/HDT/GNR or GCP/Ab/BSA	EIS [Fe(CN)_6_]^3−/4−^	rSPI2-His_6_	5 pg/mL	[25]
7.	Au/HDT/CG/Ab		hIgG	4.1 ng/L	[27]
8.	Au/Py + PyFcNHP EP/BH/SV/b-Ab	CV, DPV	PB1-F2	5 nM	[39]
9.	m-AuE/Con A/HRP/BSA + G	DPV	H9N2 virus	1 ng/mL	[40]
10.	Au MACS/CCP	EIS [Fe(CN)_6_]^3−/4−^	HA antibody	1 pg/mL	[41]
11.	Au/OT/OG/SAT + CMP-SA/BSA		H1N1 virus	-	[42]
12.	Au/MHDA/Ab/BSA		H7N1 antigen	5 μg/mL	[45]
13.	GCE/Protein A/Ab/His_6_ H5 HA/BSA		Ab against H5N1	2.1 pg/mL	[46]
14.	GCE/Protein A/Ab/BSA		PNRSV	-	[47]
15.	Au/HDT/GCP/Ab/BSA		PPV	10 pg/mL	[49]
16.	Au/HDT/GCP/Fab'/BSA		Peptides of AI H5	2.2 pg/mL	This work

*Abbreviations*: OT—octanethiol, BDDT—biotinylated dodecanethiol, NV—neutravidin, Ab—Antibody, IAV—influenza A virus, BSA—bovine serum albumin, EIS—electrochemical impedance spectroscopy, EID_50_/mL—50% Egg Infective Dose, GCE—glassy carbon electrode, SiSG—silica sol-gel, CV—cyclic voltammetry, GNPs—gold nanoparticles, HDT—1,6-hexanedithiol, GCP—gold colloidal nanoparticles, AI—avian influenza, hIgG—human immunoglobulin G, CG—colloidal gold, PB1-F2—proapoptotic protein, Py—pyrolle, PyFcNHP—1-(phthalimidylbutanoate)-1′-(N-(3-butylopyrrole)butanamide) ferrocene, EP—electropolymerization, BH—biotin hydrazide, SV—streptavidin, b-Ab—biotinylated antibody, DPV—Differential Pulse Voltammetry, m-AuE—magneto controlled home-made gold electrode, Con A—concanavalin A, HRP—horseradish peroxidase, G—glucose, MACS—microelectrode array with comb structure, CCP—coiled-coil peptide, MHDA—16-mercaptohexadecanoic acid, OG—octyl galactoside, SAT—α-2,6-sialytransferase enzyme, CMP-SA—cytidine-5′-monophospho-N-acetylneuraminic acid sodium salt-sialic acid, PNRSV—Prunus Necrotic Ringspot Virus PPV—Plum Pox Virus, His_6_ H5 HA—antigen, GNR—gold nanorods, rSPI2-His_6_—protein.
